# What do care home managers believe constitutes an ‘assessment for frailty’ of care home residents in North-West London? A survey

**DOI:** 10.1186/s12877-019-1083-5

**Published:** 2019-03-01

**Authors:** David Sunkersing, Finbarr C. Martin, Julie Reed, Maria Woringer, Derek Bell

**Affiliations:** 1 NIHR CLAHRC North West London, 369 Fulham Road, London, SW10 9NH UK; 2 Department of Primary Care and Public Health, Imperial College London, W6 8RP, London, UK; 3 King’s College London (Population Health Sciences), London, UK

**Keywords:** Frailty, Assessments, Nursing homes, Care homes, London

## Abstract

**Background:**

Frail individuals are at risk of significant clinical deterioration if their frailty is not identified and managed appropriately. Research suggests that any interaction between an older person and a health or social care professional should include an assessment for frailty. Many older care home residents are frail when admitted, but we have little knowledge of whether or how this is assessed. The aim of this paper is to understand and establish the characteristics of the reported ‘assessments for frailty’ used in care homes with nursing (nursing homes) across North-West London. This will help understand what an ‘assessment for frailty’ of care home residents mean in practice in North-West London.

**Methods:**

Telephone contact was made with every Care Quality Commission (CQC) (independent regulator of health and adult social care in England) regulated nursing home across North-West London [*n* = 87]. An online survey was sent to all that expressed interest [*n* = 73]. The survey was developed through conversations with healthcare professionals, based on literature and tested with academics and clinicians. Survey responses were analysed using descriptive statistics. The Mann-Whitney U test was used for statistical analyses.

**Results:**

24/73 nursing homes completed the survey (33%). Differences in the characteristics of reported ‘assessments for frailty’ across nursing homes were evident. Variation in high level domains assessed (physical, social, mental and environmental) was observed. Nurses were the most common professional group completing assessments for frailty, with documentation and storage being predominantly paper based. A statistically significant difference between the number of assessments used in corporate chain owned nursing homes (3.9) versus independently owned nursing homes (2.1) was observed (U = 21, *p* = .005).

**Conclusions:**

Great variation existed in the characteristics of reported ‘assessments for frailty’ in nursing homes. Our study suggests that not all physical, social, mental and environmental domains of frailty are routinely assessed: it appears that frailty is still primarily viewed only in terms of physical health. The consequences of this could be severe for patients, staff and healthcare settings. Research illustrates that frailty is a broad, multifactorial health state and, as such, an overall ‘assessment for frailty’ should reflect this.

**Electronic supplementary material:**

The online version of this article (10.1186/s12877-019-1083-5) contains supplementary material, which is available to authorized users.

## Background

By 2050, an estimated 2 billion people will be 65 years or older worldwide [1]. Though increases in life expectancy represent great advances in medicine, technology and research, the potential impact on health and social care provision poses a major policy challenge.

Moreover, since a significant proportion of individuals 65 years or older will be frail, there will be additional implications for patients, healthcare professionals and health and care settings. Frailty in individuals will need to be recognised and managed appropriately to avoid significant deterioration in their wellbeing [2]. Despite this, at present, health and social care systems are often fragmented, complicating accurate recognition, assessment and management of patients with frailty [3].

To better manage patients with frailty, there is a need to identify frailty early. However, identifying frailty early is problematic, due to its multifaceted and highly complex nature [4]. For the successful recognition, assessment and management of frailty, comprehensive assessment processes and integrated communication across settings and professional groups must be effective [5–9]. Comprehensive assessment requires holistic consideration encompassing a patient’s physical, social, mental and environmental health circumstances [9, 10]. Although a variety of assessments examine different aspects of frailty, in practice, few consider frailty across these four domains [11].

Previous studies of frail, older people have often focussed on commonly used assessments in acute care [12], such as the comprehensive geriatric assessment (CGA) – and examining their effectiveness as a measure for assessing for frailty [13, 14]. However, as many ‘assessments for frailty’ in acute care (such as the CGA) are resource intensive, a balance between completing one versus the value of doing so must be considered.

To combat potential resource and time constraints, a growing body of evidence suggests a more integrated approach towards assessing for frailty, highlighting the benefits for patients, staff and healthcare systems [15]. Indeed, the British Geriatrics Society has recommended that an ‘assessment for frailty should form part of any interaction between an older person and a health or social care professional’ [2]. Having a shared understanding of assessing for frailty could lead towards better integration across health and social care services – and ultimately better care [15].

Despite this, there is a lack of research related to assessing for frailty in non-acute care settings, particularly within care homes with nursing (nursing homes). All nursing homes must be compliant with the general standards set out by the CQC (the regulator for health and social care in England responsible for setting standards, monitoring and inspecting registered services and potentially closing services). At present, the CQC lists 13 ‘Fundamental Standards’ online which health and social care services, such as nursing homes must adhere to – and are measured against [16]. These standards cover aspects such as care and treatment, the experience level of staff and the cleanliness of premises and equipment. Notably, however, there are no mandatory requirements or recommendations regarding the specific methods or tools used to assess for frailty in residents. Accordingly, nursing homes are able to exercise a considerable degree of autonomy in assessing for frailty and may use a number of different assessments which they consider as ‘assessments for frailty’ in their home.

Assessing for frailty in nursing homes is of great importance, as many residents will be frail. Moreover, if frailty is not recognised in individuals, the associated implications on an individual’s clinical and care needs may be severe [2].

There is a consequent need to understand both how individuals are assessed for frailty in nursing homes and the characteristics that constitute the assessments used, such as their domain coverage. Discerning each nursing homes’ reported ‘assessments for frailty’ can additionally help establish a better picture of how frailty is perceived in this setting. This could ultimately support improvements in the care of frail individuals, their transfer through health and care settings and aid in integrating health and care systems.

This study aimed to establish the characteristics of the reported ‘assessments for frailty’ used in non-acute care within a specified region (North-West London) – and in a specific non-acute care setting (nursing home). The ‘assessments for frailty’ reported were nursing-home perceived. We additionally aimed to establish the frailty domains constituting the individual assessment tools used in an overall assessment for frailty. The ‘frailty domains’ (physical, social, mental, environmental) are based on work proposing a model of frailty [17].

## Methods

### Survey development: Use of a scoping study to establish perceived ‘Assessments for Frailty’

To aid in the development of the intended survey for nursing homes, a preliminary scoping study involving healthcare professionals working in non-acute care settings took place. The healthcare professionals involved included: occupational therapists, physiotherapists, pharmacists and psychiatrists. These individuals had clinical interaction with frail individuals and predominantly worked within North-West London.

The British Geriatrics Society (BGS) recommends that, ‘Any interaction between an older person and a health or social care professional should include an assessment which helps to identify if the individual has frailty’ [9]. The BGS also notes that the type of assessment used will differ when dealing with unwell versus stable individuals. At present, there is no gold standard consistently used to measure frailty, leading to many different instruments being used as ‘assessments for frailty’.

As such, using a snowball-sampling strategy, exploratory conversations with 19 different healthcare professionals took place, focussed on greater understanding of the ‘assessments for frailty’ used (if any) in non-acute settings and the nature of the mentioned assessments (e.g. its evidence base). Healthcare professionals often identified the same tools for assessing frailty. After speaking to 19 healthcare professionals, it was determined that saturation in identifying ‘assessments for frailty’ was reached. No further interviews in this scoping study took place.

In this study, we asked what ‘assessments for frailty’ were being used in non-acute care settings in North-West London. Many of the distinct frailty indexes or scales were not reported as being used in non-acute care settings in North-West London. Instead, a variety of other assessments were reported as being used, demonstrating the lack of consistency and variation in perceptions in assessing for frailty.

These assessments were reported as ‘assessments for frailty’. Many of the reported ‘assessments for frailty’ could help to identify whether an individual was suffering from one of the five common geriatric syndromes (Falls, Immobility, Delirium, Incontinence, Susceptibility to side effects of medication) consistent with the British Geriatrics Society [9], which states that the presence of one or more of these would indicate likely frailty in an individual.

Importantly, this part of the survey development enabled the views of different healthcare professionals to be incorporated into the design of the survey. This part of survey development additionally helped in the understanding of what ‘assessments for frailty’ are currently used, as well as what may constitute an assessment for frailty. Notably, there was a lack of knowledge from healthcare professionals regarding ‘assessment for frailty’ used in nursing homes, providing further justification for focussed study in this setting.

### Nursing home survey design

Following on from the preliminary scoping study, an online survey with a specific nursing home setting focus was developed using Qualtrics [18]. The survey aimed to establish the: nursing home-reported ‘assessments for frailty’ being used, the scope of health professionals using the assessment(s), the purpose(s) of the assessment(s) and documentation/storage of the assessment(s), as this may influence the ease of sharing.

As frailty can present itself and be recognised in various ways [19], participants were able to select from a number of assessments that are commonly used to assess various aspects of an individual. These options originated from the literature surrounding ‘assessments for frailty’ in use, in addition to the assessments mentioned from the healthcare professionals in the preliminary study. Including options directly derived from the preliminary study ensured that a range of perceived ‘assessments for frailty’ were available.

These assessments could be used by a nursing home to indicate impairments associated with frailty and therefore be used by a nursing home as an ‘assessment for frailty’. Participants were able to provide free text responses if no predefined options were suitable. The onus, however, was on the nursing home to report the assessments that they were using as ‘assessments for frailty’.

While the ‘assessments for frailty’ provided are not routinely defined as distinct frailty indexes or scales, our preliminary study suggested that in care home practice these may be used to help identify individuals who could be considered frail. We therefore classified these as ‘assessments for frailty’. Referring to these as ‘assessments for frailty’ in the survey was done to ensure that if the nursing homes reported their use in practice, they were reporting their use as assessments for *frailty* – and not just as an assessment for *health status*.

The process of survey design and administration was informed from literature [20, 21]. As such, the survey was tested with academics and clinicians from Imperial College London and the Collaboration for Leadership in Applied Health Research and Care North-West London (CLAHRC NWL) and checked for content, structure and clarity, thereby reinforcing the face validity of the survey. After testing, changes were made to the number of questions, phrasing and logic flow of the survey. A final version of the survey is included in the ‘Additional Files’ section of the paper (see ‘Additional file 1’).

### Recruitment

All CQC regulated nursing homes within North-West London were eligible [*n* = 87]. North-West London consists of eight boroughs, with demographic, social and economic diversity. We chose a single geography to help with an initial understanding of assessing for frailty within nursing homes, with a view to scaling up to a wider geography in later studies.

Using publicly accessible CQC information, each nursing home was contacted by telephone inviting them to participate. Information concerning the purpose and objectives of the study, confidentiality and dissemination was also given. For those who gave verbal consent to participate, an email address of the nursing home manager was requested; a link to the survey was subsequently sent to the manager. Participants were advised that the survey be completed by the manager of the nursing home, with staff input as necessary. Telephone reminder calls with each of the nursing homes who had not completed the online survey were taken at 3 weeks, 6 weeks and a final call at 18 weeks.

The survey was accessible from 30th September 2015 to 30th April 2016. No incentives were offered.

### Mapping of assessments

Assessment tools reported were mapped against frailty domains (physical, social, mental and environmental) [17].

Every effort was made to obtain a copy of the individual assessment tool to ensure mapping accuracy.

Structured and widely used assessment tools were obtained online, to help accurately identify frailty domain coverage. These were categorised as ‘Standardised’ assessment tools, characterised by their reliability, validity and consistency in test administration. The majority of these tools were also evidence-based. Contact was made with nursing homes requesting a copy of ‘in-house’ assessment tools, or those unavailable online. These tools were categorised as non-standardised – often having no clear guidance in test administration or interpretation of results. ‘Non-Standardised’ assessment tools were also identified if short, generic names were given, that could not be conclusively associated with known standardised assessment tools.

Two individuals (DS & MW) undertook the mapping exercise independently and further cross-checked with clinicians (DB & FM) to ensure accuracy.

The ownership of each nursing home was detailed in this study to further explore potential differences in ‘assessments for frailty’ used across nursing homes. For participants in this study there were two type of ownership: corporate chains (homes belonging to a large company owning multiple nursing homes with a similar aim and organisational structure) and independently owned homes (homes often belonging to one family, not part of a chain of homes).

### Statistical analysis

Comparison of the number of assessments used between corporate chain owned nursing homes and independently owned nursing homes was performed using the Mann-Whitney U test.

## Results

24/73 nursing homes completed the survey (33%). We received responses from all 8 boroughs within North-West London.

For clarity in this paper, each individual ‘assessment for frailty’ reported will be referred to as an ‘assessment tool’. A nursing home may have reported multiple ‘assessment tools’ which forms their overall ‘assessment for frailty’.

### Assessment tools reported

In total, 77 individual assessment tools were reported, composed of standardised (43) and non-standardised assessment tools (34) (Table 1).

**Table 1:** Standardised Assessment Tools Reported

Assessment Tool	Type	Quantity	% of Total Assessment Tools Reported
Barthel Index of Activities of Daily Living	Standardised	9	11.7
Mini Mental State Examination (MMSE)	Standardised	8	10.4
Falls Efficacy Scale International (FES)	Standardised	7	9.1
The Home Falls and Accidents Screening Tool (HOME FAST)	Standardised	5	6.5
Waterlow	Standardised	3	3.9
Clifton Assessment Procedures for the Elderly (CAPE)	Standardised	2	2.6
Falls Risk Assessment Scale for the Elderly (FRASE)	Standardised	2	2.6
West London Mental Health NHS Trust Occupational Therapy Functional Assessment	Standardised	2	2.6
Falls Efficacy Scale International (Short Form FES)	Standardised	1	1.3
Beck Depression Inventory	Standardised	1	1.3
Central London Community Hospitals (CLCH) Multifactorial Falls Assessment	Standardised	1	1.3
Gait Assessment (Tinetti Performance Oriented Assessment of Mobility)	Standardised	1	1.3
Malnutrition Universal Screening Tool (MUST)	Standardised	1	1.3

13 unique standardised assessment tools were reported; 18 non-standardised assessment tools were reported.

We could not conclude whether non-standardised tools reported were unique or not. This occurred when participants detailed a non-standardised tool with an identical generic name to other participants. For example, there were 4 non-standardised assessment tools which participants detailed only as ‘Falls’ or ‘Falls Assessment’. In the table below (Table 2), these are categorised under the assessment tool heading ‘Falls’.

**Table 2:** Non-Standardised Assessment Tools Reported

Assessment Tool	Type	Quantity	% of Total Assessment Tools Reported
Falls	Non-Standardised	4	5.2
Mood / Depression Assessment Questionnaire	Non-Standardised	4	5.2
Nutrition	Non-Standardised	4	5.2
Pre-Admission	Non-Standardised	3	3.9
Risk Assessment	Non-Standardised	3	3.9
Environmental	Non-Standardised	2	2.6
Medication	Non-Standardised	2	2.6
Mobility Assessment	Non-Standardised	2	2.6
Pre and Post Assessment (1 tool)	Non-Standardised	1	1.3
Activities	Non-Standardised	1	1.3
Continuing Tool	Non-Standardised	1	1.3
Daily Living	Non-Standardised	1	1.3
Dependency Tool	Non-Standardised	1	1.3
Eyesight	Non-Standardised	1	1.3
Functional Assessment	Non-Standardised	1	1.3
Memory and Dementia Screening	Non-Standardised	1	1.3
Mental Capacity Assessment	Non-Standardised	1	1.3
Overall Health	Non-Standardised	1	1.3

### Assessment tool use by nursing home

An average of 3.2 assessment tools (Range: 1–6) were used per nursing home. A statistically significant difference in the number of assessments used in corporate chain owned nursing homes (Assessment tool range: 1–6; Average no. of assessment tools: 3.9) versus independently owned nursing homes (Assessment tool range: 1–3; Average no. of assessment tools: 2.1) was observed (U = 21, *p* = .005).

Thirteen nursing homes used more standardised than non-standardised assessment tools. 7 nursing homes used more non-standardised assessment tools than standardised assessment tools. Four homes used an equal number of standardised and non-standardised assessment tools.

### Domain coverage

**Fig. 1:**
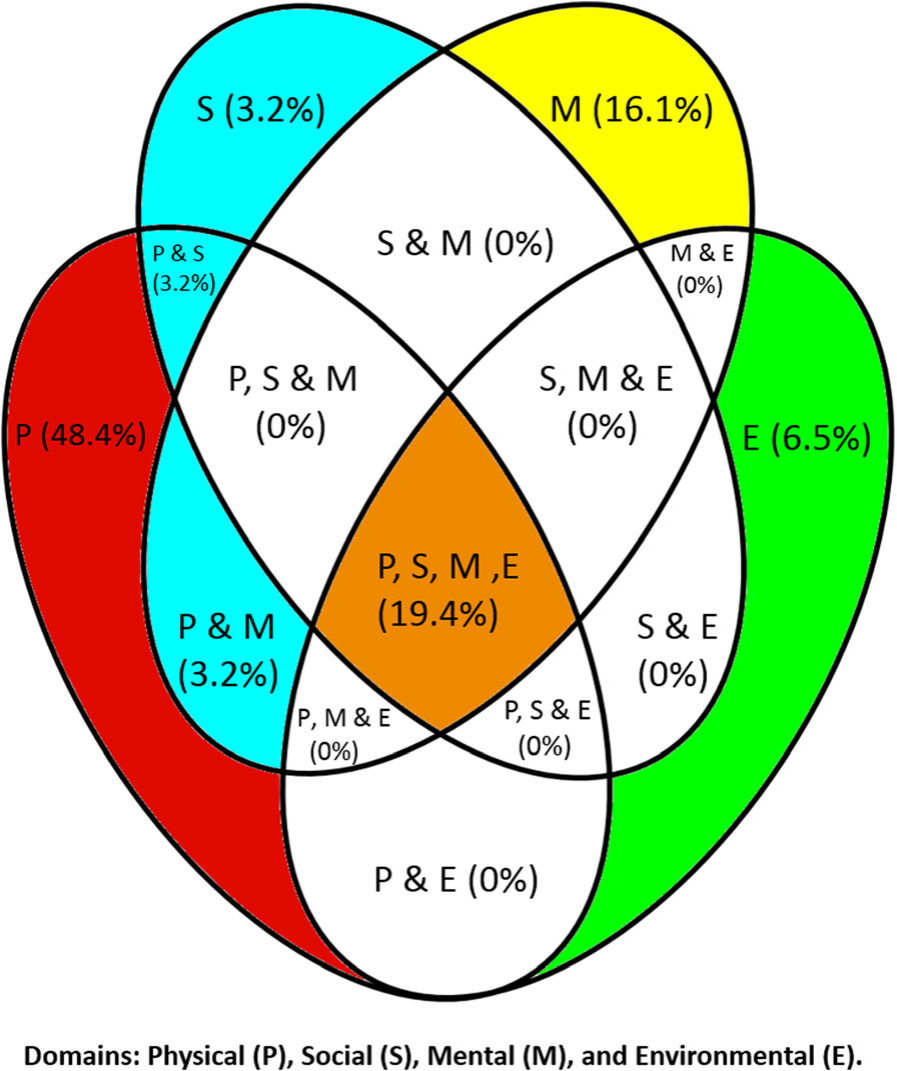
Venn Diagram Illustrating Domain Coverage of Individual Frailty Assessment Tools Reported from All Nursing Homes

Reported assessment tools most frequently measured the physical domain (48.4%) (e.g. mobility, falls assessments), mental health domain (16.1%) (e.g. mood and depression assessments), environmental domain (6.5%) (e.g. Home Falls and Accidents Screening Tool) and social domain (3.2%) (e.g. Social activities assessment). 19.4% of reported tools covered all domains. Some tools covered both physical and social domains (3.2%), in addition to both physical and mental domains (3.2%). This is illustrated in Fig. 1 below:

### Health professionals using the assessment tools

The health professionals stated to be using the assessment tools were: nurse (54.2%), doctor (14.6%), occupational therapist (8.3%), manager (7.3%), nurse assistant (5.2%), physiotherapist (5.2%), MDT (Multi-Disciplinary Team) (4.2%) and community psychiatric nurse (1.0%). Where MDT was reported, no further details were given.

### Purpose of assessment

Assessment tools were used: to help inform clinical decision making (47.4%), for resource allocation (26.8%), for funding (7.2%), for care planning (6.2%) and 12.4% classified use as ‘Other’.

### Documentation and storage of assessment tools (electronic or paper based)

Details of storage was received from 21 of the 24 nursing homes (88%). The majority of respondents stated that assessment tools were paper based (85.9%) rather than electronic (14.1%).

### Non-participants: Ownership

The 14/87 eligible nursing homes declining to participate after initial telephone contact were: 7 owned by corporate chains; 7 independently owned. Of the 49/73 nursing homes sent the online survey after expressing interest, but not completing the online survey, 26 were owned by corporate chains; 23 independently owned. Some nursing homes that did not complete the online survey belonged to corporate chains 1, 2, 3, 4, 6, 9 and 13 – corporate chains owning nursing homes that did complete the online survey (Table 3).

**Table 3:** Assessment Tool use by Nursing Home

Nursing Home (Anonymised)	Ownership	No. of Standardised Assessment Tools	No. of Non-Standardised Assessment Tools	Total No. of Assessment Tools Reported
A	Corporate Chain 1	5	1	6
B	Corporate Chain 2	0	6	6
C	Corporate Chain 3	5	0	5
D	Corporate Chain 4	4	1	5
E	Corporate Chain 5	4	1	5
F	Corporate Chain 6	1	3	4
G	Corporate Chain 7	2	2	4
H	Corporate Chain 8	1	3	4
I	Corporate Chain 9	2	2	4
J	Corporate Chain 6	0	4	4
K	Corporate Chain 11	0	3	3
L	Corporate Chain 3	3	0	3
M	Corporate Chain 13	2	0	2
N	Corporate Chain 3	2	0	2
O	Corporate Chain 15	1	0	1
P	Independent	3	0	3
Q	Independent	0	3	3
R	Independent	0	3	3
S	Independent	2	0	2
T	Independent	1	1	2
U	Independent	1	1	2
V	Independent	2	0	2
W	Independent	1	0	1
X	Independent	1	0	1

## Discussion

To our knowledge, this is the first study that has looked at the characteristics of the reported ‘assessments for frailty’ used in nursing homes across North-West London. A single geographic focus (North-West London) provides an initial understanding of ‘assessments for frailty’ used in nursing homes, strengthening our knowledge on how frailty is currently perceived in this setting.

The results illustrated more reported standardised assessment tools (43) than non-standardised assessment tools (34) overall. Similarly, a greater number of nursing homes used more standardised assessment tools in their reported assessment for frailty than non-standardised assessment tools. Although not recent, the single assessment process (SAP) [22], whereby thorough, accurate and recognisable assessments were encouraged nationally, could provide reasoning behind the relatively high usage of standardised assessment tools. Taking the core principles from the SAP led to the production of the Common Assessment Framework (CAF), which further encouraged information capture in a standardised approach [23]. Standardised assessment tools offer a consistency, validity through an evidence base and transferability across several healthcare settings. A historical encouragement within health and social care services through recommendations such as the SAP and CAF could explain the marginally higher use of standardised assessment tools versus non-standardised assessment tools reported in this study.

Our study additionally indicated that 9 nursing homes used a combination of non-standardised and standardised assessment tools – and 5 nursing homes used only non-standardised assessment tools. Several studies have suggested that the use of non-standardised assessment tools are not uncommon - and perhaps related to skill, time and motivation [24]. Despite this, further exploration (e.g. through observation) is required to fully understand the reasoning behind these assessment choices – especially when many valid and reliable standardised assessment tools are in existence.

We found overall variation in the number of ‘assessments for frailty’ used. Our study indicated a statistically significant difference in the number of assessments used in corporate chain owned nursing homes (higher) versus independently owned nursing homes, suggesting different levels of comprehensiveness in assessing for frailty between the two. One corporate chain of nursing homes (Corporate chain 6) used the same number of ‘assessments for frailty’ in both nursing homes they owned. Conversely, one corporate chain owning three nursing homes (Corporate chain 3), responded with varying numbers of ‘assessments for frailty’ used (2, 3 and 5). Overall, there was greater variation in the number of ‘assessments for frailty’ reported in nursing homes owned by a corporate chain (1–6) than in independently owned nursing homes (1–3). It is possible that this represents variation in each nursing home’s interpretation of what constitutes an ‘assessment for frailty’. Nevertheless, we question why so much variation exists in the number of ‘assessments for frailty’ used across North West London, but also, why variation exists within nursing homes belonging to the same corporate chain.

Another key finding was that the predominant domain assessed related to physical assessment (physical domain), followed by assessments pertaining to an individual’s mental health (mental domain). Arguably, these domains are the most visible domains that frailty affects, perhaps explaining the dominance of these two domains in the assessment tools used. The lower number of assessment tools covering the social and environmental domains could be directly related to the nursing homes’ perceived ability to have sufficient control over these aspects of care. Alternatively, it could be that fewer assessments exist, or are known, that sufficiently cover social and environmental domains. These results, however, perhaps represent a deviation from research led recommendations for holistic assessments in clinical settings. Moreover, this finding could illustrate that frailty is not being recognised or assessed for as comprehensively as it should be. Research has demonstrated that if frailty is unrecognised, there may be severe consequences to the health and wellbeing of the individual [2].

The finding that the majority of assessments were being used by nurses (54.2%) and doctors (14.6%) is a somewhat expected result. All nursing homes in the UK are required to have a registered nurse on duty at all times. Nevertheless, our results also signify that managers and nurse assistants were using the assessments, potentially indicating staff shortages and increases in the complexity of demand – a challenge noted in the latest State of Care report [25]. We did not, however, verify whether the managers were also qualified nurses.

Our survey revealed the most popular reason explaining the assessment purpose was to help inform clinical decision making (47.4%) and for resource allocation (26.8%). This finding is supported with several references detailing the importance of recognising the different degrees of frailty – and understanding that this will impact the clinical course and resource requirements [26].

The majority of respondents stated that assessment tools were paper based (85.9%) rather than electronic (14.1%). Recent studies have suggested no difference in the quality of documentation content from an electronic system versus a paper-based system within a nursing home setting [27]. However, evidence from research in healthcare environments such as acute care has indicated that an electronic system can bring improved advantages over that of a paper based system, contributing to improved efficiency, quality of care and patient safety [28, 29]. While there may be cost, time and training implications in the implementation of an electronic system, it is possible that the advantages documented in acute care may also carry forward into the nursing home setting.

Despite the presence of ‘assessments for frailty’ in all nursing homes, the variation in the characteristics of the assessments will greatly influence the extent and effectiveness to which frailty is recognised and managed. This variation is a potential barrier to the integration and ease of communication between different healthcare settings and professionals.

Evidence from a recent systematic review highlighted that many long term care facilities such as nursing homes were reluctant to participate in research – and some were prohibited from participating due to corporate policies [30]. This may in part explain the response rate and reasoning behind the 14 non-participating nursing homes that declined to take part in the study following initial telephone contact.

This study found a similar proportion of corporate chain owned versus independently owned nursing homes who were sent, but did not complete, the online survey (26 corporate chain owned nursing homes; 23 independently owned). Further research is required to fully understand why these nursing homes did not participate. It is possible that the nursing homes staff did not complete the survey because of time or resource pressures [25, 31]. Some corporate chains owned both participating and non-participating nursing homes, potentially due to a lack of clarity regarding research participation policies, or participation being at the discretion of each home.

Observational longitudinal studies could aid in reducing response bias and gain greater accuracy in assessment use. They could also help clarify individual nursing home expertise, in addition to ascertaining the time and cost implications of assessments used. This could help explain the extent of variation that exists in the characteristics of the reported assessments used.

## Limitations

Survey response rate was a limitation to our study, though the response rate (33%) is comparable to that achieved in other studies that used an online survey approach [32]. Follow-up requests via telephone at three stages aimed to maximise the number of participants. Response bias may have influenced responses given, perhaps reflecting what would be done ideally rather than what happens day-to-day (i.e. ‘work-as-imagined’ vs ‘work-as-done’). The survey may not have captured the ‘assessments for frailty’ used by visiting health professionals. The preliminary scoping study, whereby the ‘assessments for frailty’ used by a range of professionals were captured and used in the nursing homes survey aimed to mitigate this. This study examined assessment use in North-West London; the results are not necessarily reflective of nursing home practices in a wider geographical area.

## Conclusions

Our study suggests that not all four domains of frailty were being assessed in individuals in nursing homes across North-West London - and suggest that frailty is still primarily viewed as a condition of deteriorated physical health. This is a major issue, as it suggests that there are frail individuals in this setting whose clinical and care needs have not been appropriately recognised or managed. The consequences of this lack of recognition could be severe for patients, staff and healthcare settings, with frailty only being recognised when an individual experiences an emergency, such as a fall. Research illustrates that frailty is a broad, multifactorial health state and, as such, an overall assessment for frailty should reflect this.

## Additional file

Additional file 1: Assessments for Frailty in Nursing Homes. This is the online survey that was sent to the nursing homes using Qualtrics. (PDF 301 kb)

## Abbreviations

BGS: British Geriatrics Society
CAF: Common Assessment Framework
CGA: Comprehensive geriatric assessment
CLAHRC NWL: Collaboration for Leadership in Applied Health Research and Care North-West London
CQC: Care Quality Commission
MDT: Multidisciplinary team
SAP: Single assessment process

## References

[1] Department of Economic and Social Affairs. United Nations Secretariat. World Population Ageing. United Nations. 2014. [Online] Available at: http://www.un.org/en/development/desa/population/publications/pdf/ageing/WPA2015_Report.pdf. Accessed 13 Jan 2016.

[2] British Geriatrics Society. Fit for Frailty Part 2 - Developing, commissioning and managing services for people living with frailty in community settings - a report from the British Geriatrics Society and the Royal College of General Practitioners. 2015. [Online]. Available at: https://www.bgs.org.uk/sites/default/files/content/resources/files/2018-05-23/fff2_full.pdf. Accessed 9 July 2018.

[3] House of Commons. The Health Committee. Social Care. Fourteenth Report of Session 2010–2012. UK Parliament. 2012. [Online] Available at: http://www.publications.parliament.uk/pa/cm201012/cmselect/cmhealth/1583/1583.pdf. Accessed 5 May 2015.

[4] Bergman H, Ferrucci L, Guralnik J (2007). Frailty: an emerging research and clinical paradigm-issues and controversies. J Gerontol Ser A Biol Med Sci.

[5] Royal College of General Practitioners and British Geriatrics Society. Integrated care for older people with frailty. Innovative approaches in practice. 2016. [Online]. Available at: https://www.bgs.org.uk/sites/default/files/content/resources/files/2018-10-09/RCGP-Integrated-care-for-older-people-with-frailty-2016.pdf. Accessed 15 Jan 2018.

[6] National Institute for Health Research. Comprehensive Care. Older people living with frailty in hospitals. 2017. [Online]. Available at: http://www.dc.nihr.ac.uk/themed-reviews/Comprehensive-Care-final.pdf. Accessed 15 Jan 2018.

[7] Kodner D, Kyriacou C (2000). Fully integrated care for frail elderly: two American models. Int J Integr Care.

[8] Wilson A, Baker R, Bankart J et al. Establishing and implementing best practice to reduce unplanned admissions in those aged 85 years and over through system change [establishing system change for admissions of people 85+ (ESCAPE 85+)]: a mixed-methods case study approach. Southampton: NIHR Journals Library; 2015. (Health Services and Delivery Research, No. 3.37.) 26334078

[9] British Geriatrics Society. Fit for Frailty Part 1 - Consensus best practice guidance for the care of older people living in community and outpatient settings - A report from the British Geriatrics Society in association with the Royal College of General Practitioners and Age UK. 2014. [Online]. Available at: https://www.bgs.org.uk/sites/default/files/content/resources/files/2018-05-23/fff_full.pdf. Accessed 17 Jan 2019.

[10] Welsh TJ, Gordon AL, Gladman JR (2014). Comprehensive geriatric assessment – a guide for the non-specialist. Int J Clin Pract.

[11] Clegg A, Young J, Iliffe S, Olde Rikkert M, Rockwood K (2013). Frailty in elderly people. Lancet.

[12] Quinn TJ, McArthur K, Ellis G, Stott DJ (2011). Functional assessment in older people. BMJ (Clin Res ed.).

[13] Ellis G, Whitehead MA, O’Neill D, Langhorne P, Robinson D (2011). Comprehensive geriatric assessment for older adults admitted to hospital. Cochrane Database Syst Rev.

[14] Ekerstad N, Karlson BW, Dahlin Ivanoff S, Landahl S, Andersson D, Heintz E, Alwin J (2017). Is the acute care of frail elderly patients in a comprehensive geriatric assessment unit superior to conventional acute medical care?. Clin Interv Aging.

[15] NHS England. Safe, compassionate care for frail older people using an integrated care pathway: Practical guidance for commissioners, providers and nursing, medical and allied health professional leaders. 2014. Available at: www.england.nhs.uk/wp-content/uploads/2014/02/safe-comp-care.pdf. Accessed 15 Jan 2018.

[16] CQC. 2019. The fundamental standards [Online]. Available at: https://www.cqc.org.uk/what-we-do/how-we-do-our-job/fundamental-standards. Accessed 9 Jan 2019.

[17] Soong J, Bailey A, Bell D. Re. functional assessment in older people. BMJ. 2013 [Online] Available at: http://www.bmj.com/content/343/bmj.d4681/rr/645724. Accessed 7 Nov 2017.

[18] The data for this paper was generated using Qualtrics software, Version 2.5 of Qualtrics. Copyright © [2018] Qualtrics. Qualtrics and all other Qualtrics product or service names are registered trademarks or trademarks of Qualtrics, Provo. https://www.qualtrics.com.

[19] Turner G, Clegg A (2014). Best practice guidelines for the management of frailty: a British geriatrics society, age UK and Royal College of general practitioners report. Age Ageing.

[20] McColl E, Jacoby A, Thomas L, Soutter J, Bamford C, Steen N, Thomas R, Harvey E, Garratt A, Bond J. Design and use of questionnaires: a review of best practice applicable to surveys of health service staff and patients. Health Technol Assess. 2001;5(31):1–256. 10.3310/hta5310 11809125

[21] Kelley K, Clark B, Brown V, Sitzia J (2003). Good practice in the conduct and reporting of survey research. Int J Qual Health Care.

[22] Department of Health. National Service Framework for Older People. [pdf]. London: Department of Health; 2001. Available at https://assets.publishing.service.gov.uk/government/uploads/system/uploads/attachment_data/file/198033/National_Service_Framework_for_Older_People.pdf. Accessed 17 May 2018.

[23] Department of Health. Common Assessment Framework for Adults. [pdf]. London: Department of Health; 2009. Available at https://www.networks.nhs.uk/nhs-networks/common-assessment-framework-for-adults-learning/archived-material-from-caf-network-website-pre-april-2012/documents-from-discussion-forum/CAF%20Full%20Consultation-dh_093715.pdf. Accessed 17 June 2018.

[24] Wales K, Clemson L, Lannin N, Cameron I (2016). Functional assessments used by occupational therapists with older adults at risk of activity and participation limitations: a systematic review. PLoS One.

[25] Care Quality Commission. The state of health care and adult social care in England 2016/17. 2017. [Online]. Available at: https://www.cqc.org.uk/sites/default/files/20171123_stateofcare1617_report.pdf. Accessed 15 Jan 2018.

[26] Rockwood K, Theou O, Mitnitski A (2015). What are frailty instruments for?. Age Ageing.

[27] Wang N, Yu P, Hailey D (2015). The quality of paper-based versus electronic nursing care plan in Australian aged care homes: a documentation audit study. Int J Med Inform.

[28] Eden KB, Messina R, Li H, Osterweil P, Henderson CR, Guise JM (2008). Examining the value of electronic health records on labor and delivery. Am J Obstet Gynecol.

[29] Pollak VE, Lorch JA (2007). Effect of electronic patient record use on mortality in end stage renal disease, a model chronic disease: retrospective analysis of 9 years of prospectively collected data. BMC Med Inform Decis Mak.

[30] Lam HR, Chow S, Taylor K, Chow R, Lam H, Bonin K, Rowbottom L, Herrmann N (2018). Challenges of conducting research in long-term care facilities: a systematic review. BMC Geriatr.

[31] Burns DJ, Hyde PJ, Killett AM (2016). How financial cutbacks affect the quality of jobs and care for the elderly. ILR Rev.

[32] Nulty D (2008). The adequacy of response rates to online and paper surveys: what can be done?. Assess Eval High Educ.

