# Supplementary material for: What do care home managers believe constitutes an ‘assessment for frailty’ of care home residents in North-West London? A survey
Source: BMC Geriatr. 2019 Mar 1;19:62. doi: 10.1186/s12877-019-1083-5 (PMC6397475; doi:10.1186/s12877-019-1083-5)
Supplement: Supplementary file 1 — Assessments for Frailty in Nursing Homes. This is the online survey that was sent to the nursing homes using Qualtrics. (PDF 301 kb) [file 12877_2019_1083_MOESM1_ESM.pdf]

Assessments for Frailty in Nursing Homes

Which structured assessments for frailty are used? *Please select as many options that apply. If partial assessments are being used, please specify this under 'Other'.*

❑ Central London Community Hospitals (CLCH) Multifactorial Falls Assessment
❑ Timed Up and Go
❑ 30 Second Sit to Stand
❑ Falls Efficacy Scale International (FES)
❑ Falls Efficacy Scale International (Short Form FES)
❑ BERG Balance Scale
❑ Short Form BERG Balance Scale (7-Item version)
❑ Balance Assessment (Tinetti Performance Oriented Assessment of Mobility)
❑ Gait Assessment (Tinetti Performance Oriented Assessment of Mobility)
❑ The Home Falls and Accidents Screening Tool (HOME FAST)
❑ Cohen-Mansfield Agitation Inventory
❑ Montreal Cognitive Assessment / MoCA-BLIND
❑ Screening Tool for Older Peoples' Inappropriate Treatment (STOPIT) review
❑ West London Mental Health NHS Trust Occupational Therapy (WLMHT O.T.) Functional
❑ Assessment
❑ Addenbrooke's Cognitive Examination (ACE-R)
❑ Mood / Depression Assessment Questionnaire
❑ Beck Depression Inventory
❑ Neuropsychiatric Inventory
❑ Mini Mental State Examination (MMSE)
❑ Hospital Anxiety and Depression Scale
❑ Electronic Frailty Index (eFI)
❑ Assessment of Motor and Process Skills (AMPS)
❑ Barthel Index of Activities of Daily Living
❑ Other (Please fill in the blank) (Text-Entry Box)
❑ Other (Please fill in the blank) (Text-Entry Box)
❑ Other (Please fill in the blank) (Text-Entry Box)
❑ No assessments for frailty are used

(For each option reported to be in use, the following questions were asked. 'Assessment Example' has been used to represent an option reported to be in use)

Which health professionals are using the 'Assessment Example'? *Please list as many that apply.*

❑ Doctor (Please specify type) (Text-Entry Box)
❑ Nurse (Please specify type) (Text-Entry Box)
❑ Physiotherapist
❑ Dietician
❑ Occupational Therapist
❑ Nurse Assistant
❑ Admin/Support Employees
❑ Other (Please specify) (Text-Entry Box)

Why is the ‘Assessment Example’ being used? *For example: in clinical decision making / resource allocation / end of life planning either by a single profession or as part of a wider MDT group or colleagues.*

(Text-Entry Box)

How are the responses to the ‘Assessment Example’ stored? *Please select only one option.*

❑ Paper
❑ Electronically (if so, what database?) (Text-Entry Box)
❑ Other (please specify) (Text-Entry Box)
❑ Don't Know

We would appreciate it if you could upload a copy of the assessments for frailty you mentioned. Please could you state whether this is possible.

❍ Yes - Assessments will be uploaded here
❍ No - Assessments cannot be uploaded here, but a site visit can be arranged to view assessments used
❍No - Assessments cannot be uploaded here and a site visit cannot be arranged
